# Electrode placement on the forearm for selective stimulation of finger extension/flexion

**DOI:** 10.1371/journal.pone.0190936

**Published:** 2018-01-11

**Authors:** Xueliang Bao, Yuxuan Zhou, Yunlong Wang, Jianjun Zhang, Xiaoying Lü, Zhigong Wang

**Affiliations:** 1 State Key Laboratory of Bioelectronics, Southeast University, Nanjing, People’s Republic of China; 2 Co-innovation Center of Neuroregeneration, Nantong University, Nantong, People’s Republic of China; 3 Institute of RF- & OE-ICs, Southeast University, Nanjing, People’s Republic of China; Tokai University, JAPAN

## Abstract

It is still challenging to achieve a complex grasp or fine finger control by using surface functional electrical stimulation (FES), which usually requires a precise electrode configuration under laboratory or clinical settings. The goals of this study are as follows: 1) to study the possibility of selectively activating individual fingers; 2) to investigate whether the current activation threshold and selective range of individual fingers are affected by two factors: changes in the electrode position and forearm rotation (pronation, neutral and supination); and 3) to explore a theoretical model for guidance of the electrode placement used for selective activation of individual fingers. A coordinate system with more than 400 grid points was established over the forearm skin surface. A searching procedure was used to traverse all grid points to identify the stimulation points for finger extension/flexion by applying monophasic stimulation pulses. Some of the stimulation points for finger extension and flexion were selected and tested in their respective two different forearm postures according to the number and the type of the activated fingers and the strength of finger action response to the electrical stimulation at the stimulation point. The activation thresholds and current ranges of the selectively activated finger at each stimulation point were determined by visual analysis. The stimulation points were divided into three groups (“Low”, “Medium” and “High”) according to the thresholds of the 1^st^ activated fingers. The angles produced by the selectively activated finger within selective current ranges were measured and analyzed. Selective stimulation of extension/flexion is possible for most fingers. Small changes in electrode position and forearm rotation have no significant effect on the threshold amplitude and the current range for the selective activation of most fingers (*p* > 0.05). The current range is the largest (more than 2 mA) for selective activation of the thumb, followed by those for the index, ring, middle and little fingers. The stimulation points in the “Low” group for all five fingers lead to noticeable finger angles at low current intensity, especially for the index, middle, and ring fingers. The slopes of the finger angle variation in the “Low” group for digits 2~4 are inversely proportional to the current intensity, whereas the slopes of the finger angle variation in other groups and in all groups for the thumb and little finger are proportional to the current intensity. It is possible to selectively activate the extension/flexion of most fingers by stimulating the forearm muscles. The physiological characteristics of each finger should be considered when placing the negative electrode for selective stimulation of individual fingers. The electrode placement used for the selective activation of individual fingers should not be confined to the location with the lowest activation threshold.

## Introduction

Functional electrical stimulation (FES) is a clinical therapeutic technique that can help paralyzed patients to recover limb motor function lost due to stroke or spinal cord injury [1]. The electrical stimulation pulses act on the nerve or the target muscle through surface [2–4] or implanted electrodes [5] to induce artificial limb movements. Surface FES is widely accepted by patients because it is noninvasive and convenient.

The therapeutic efficacy of FES on the muscle function rehabilitation of a paretic limb depends on several factors, including the time point of interventional therapy, the intensity of FES, and the amount of training [6]. However, many factors, such as muscle fatigue, discomfort, and poor muscle selectivity, limit the long-term use and effectiveness of FES. Previous studies have shown that the size and location of the surface electrode are two of the factors associated with discomfort and muscle selectivity [6–9]. The optimal electrode size depends on the target muscle. Small electrodes are more precise than larger ones for the selective activation of forearm muscles. However, accurate positioning of a small electrode may require more time than a larger electrode to test over the skin surface of the target muscle [10]. The location of the stimulating electrode is critical for eliciting the desired muscle contractile response. Positioning the electrode close to the sensory nerve may lead to activation of skin surface receptors and cause discomfort. Consequently, patients may refuse such treatment [6]. Therefore, the electrode location is an urgent issue in clinical applications of body surface electrical stimulation [11–16]. The Handmaster^™^ system has multiple surface electrodes attached to the splint of an FES device, allowing users to easily operate it. However, it is difficult to change the position of the stimulating electrodes. For the ETHZ-ParaCare^™^ nerve prosthesis, it takes approximately 10 min to correct the electrodes to the right positions [17]. Problems associated with these devices include the somewhat limited limb-action selectivity and the time-consuming positioning of the electrodes. Therefore, quickly and accurately positioning the surface electrodes in the correct locations is an important prerequisite for the effective application of surface FES.

After effective treatments with the currently available FES, patients can perform some simple functional tasks, including gripping and wrist extension [2, 4]. However, most current electrical stimulators cannot enable patients to make the fine finger movements that are commonly lost in stroke patients. Achieving fine control of finger movements first requires the identification of the stimulation points for each finger. Several groups have previously used pen electrodes to locate muscle motor points [18–19]. Theoretically, the motor point is an optimal stimulus position [20]. In previous studies, the stimulation (negative) electrodes were placed on the optimal motor points [10] or in location(s) with the lowest activation thresholds [21] to achieve the selective control of individual fingers. Here, we assumed that points with higher thresholds than the motor point could also serve as options for negative electrode placement if desired movements could be effectively caused without reports of discomfort during the stimulation. Thus, it is highly meaningful to determine the locations of the stimulation points at which the extension/flexion of individual fingers could be activated.

At the physiological level, there is positive theoretical support for fine finger movement control. The superficial multi-tendoned muscles, such as the extensor digitorum communis (EDC) and flexor digitorum superficialis (FDS), can selectively activate digits 2–5. The single-tendoned muscles, such as the extensor indicis (EI), extensor digiti minimi (EDM), and flexor pollicis longus (FPL), also activate or assist in the extension or flexion of specific fingers [10, 21–23].

Several groups have attempted to selectively control individual fingers with surface FES. Keller et al. observed that the flexion of the middle and ring fingers could be selectively stimulated in all participants by applying FES to the finger flexor muscles [24]. By applying FES to the EDC, FDS, and the thenar muscles, Westerveld et al. found that it was possible to selectively stimulate 3 or 4 fingers, and the selective stimulation of extension of the middle, index, and ring fingers and flexion of the thumb could be achieved in most participants [10]. These studies confirmed the possibility of selective activation of individual fingers via the application of FES to the forearm muscles.

To selectively stimulate a single finger, the distribution of stimulation points for each finger should be determined first. However, there are currently no reports on the distribution of stimulation point locations or maps for the selective stimulation of an individual finger. Westerveld et al. sought stimulation points for a specific finger in a 4×6 grid drawn on the EDC muscle [10]. However, that method could not be applied to everyone, because the position of the EDC muscle is not easily identified. Then, the activation threshold and selective range of each stimulation point to the finger were measured. Lawrence et al. found that the optimal motor point shifted with forearm rotation (pronation and supination) [21].

A relative displacement of the stimulation point may result in a change in the activation threshold. Thus, we attempted to explore whether small shifts in the position of the negative electrode in a specific forearm posture and the displacement of the stimulation point in different forearm postures affected the activation threshold and selective range of each individual finger. The assumption that the points with high activation thresholds should be used to selectively stimulate individual fingers was validated by dividing the stimulation points into three groups, measuring the angle of the stimulated finger in the selective current range, and analyzing the trend in angle variation. By validating this hypothesis, we may obtain a theoretical guide for the placement of negative electrodes in order to selectively stimulate individual fingers.

The purpose of this study can be summarized as follows:

1. To determine the location of the stimulation points for finger extension and flexion by applying a certain current intensity to the grid points in the forearm coordinate system and to study the possibility and range of selective activation of individual fingers using surface FES;
2. To investigate whether small changes in electrode placement in a forearm posture and the relative displacement of the stimulation point (resulting from rotation of the forearm) influence the activation threshold and selective range of each individual finger;
3. To develop theoretical guidance for electrode placement used for the selective activation of individual fingers by studying whether the finger activation threshold influences the trend of finger angle variation.

## Methods

### Participants

Eight healthy young volunteers (7 male and 1 female, aged 23–30 years, right-handed) participated in this experiment. They gave written informed consent after the experiment was approved by the Southeast University ethics committees. One week before the experiment, each participant was instructed not to perform strenuous movements associated with the upper limbs. The right forearm was stimulated during the tests.

### Experimental setup

#### Electrical stimulation and electrodes

A Master-9^™^ programmer neuromuscular stimulator (AMPI Company, Jerusalem, Israel) with an isolator was used to generate monophasic negative pulse trains with constant parameters (pulse width and frequency). The maximum output current amplitude was 10 mA. Round hydrogel electrodes, 2.2 cm in diameter, were used as the negative electrodes, and rectangular hydrogel electrodes of 4×4 cm were used as the positive electrode.

#### Finger angle measurement

A self-developed data acquisition system, including a PC with appropriate software and an inertial measurement unit (IMU) sensor module incorporating a micro-controller unit (MCU) chip (STM32F051K8U6) and an MPU9250 sensor, was used to record and store the finger action angle data. Then, the 3D angle values, including pitch, yaw and roll, were estimated by a quaternion-based Kalman filter algorithm in the MCU and sent in real time through the serial port to the software on the PC. Generally, the angle between the metacarpophalangeal joint (MCP) and the back of the hand is in the range of 70~90°. The range of motion (ROM) of the proximal interphalangeal joint (PIP) is lower than that of the distal interphalangeal joint (DIP) and greater than that of the MCP. It is more accurate in response to the FES than the PIP and DIP joints. Therefore, the IMU sensor was mounted using foam adhesive on the place between the PIP and DIP of the fingers to monitor the PIP joint movement of the digits (extension and flexion). For the thumb, both angles of the DIP and PIP were measured. The IMU modular sensor had an angular resolution of 0.01° and a data sampling rate of 100 Hz. The sum of the movement angles of the finger in 3D space were calculated as follows:

$$Angle=\sqrt{roll^{2}+pitch^{2}+yaw^{2}}$$ (1)

#### Coordinate system for electrode positioning

A coordinate system with more than 400 grid points was established over the forearm skin surface. The length of the forearm is divided into 12 equally spaced segments from the elbow crease to the wrist crease. This cross-individual measurement method is similar to the bone proportional *cun* (B-c*un*) method used for locating acupuncture points in Chinese medicine [25]. For each participant, the distance between two adjacent grid points is scaled according to the length of the participant’s forearm, as the points are defined and drawn relative to anatomical landmarks (as given in Table 1).

**Table 1:** Forearm length and actual size corresponding to 2L (1/12 of the forearm length) for each of the 8 participants.

Participants	Forearm length (cm)	2L (cm)	L (cm)
A	25.50	2.13	1.06
B	25.80	2.15	1.08
C	23.80	1.98	0.99
D	25.90	2.16	1.08
E	25.70	2.14	1.07
F	25.00	2.08	1.04
H	26.00	2.16	1.08
I	25.00	2.08	1.04

**Fig 1:**
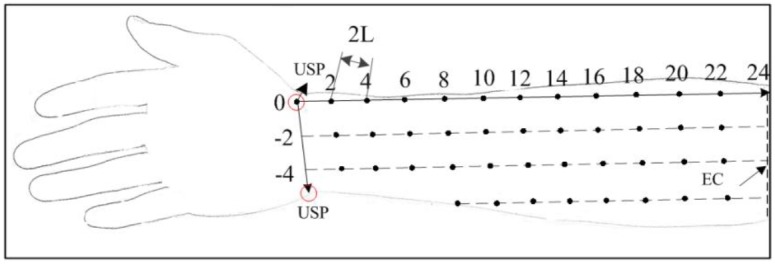
Coordinate system established for the determination of stimulation points of the finger extension and flexion.

The inter-grid distance between points in different columns is equal to the distance between two rows in order to make the coordinate system more standard, although the circumference of the forearm decreases from the proximal to distal end. The end of the radial distal wrist crease in the forearm in the supine posture has been defined as the origin, the medial distal wrist crease has been defined as the positive x-axis, the dorsal wrist crease toward the ulna styloid process (USP) has been defined as the negative x-axis, and the line connecting the wrist crease and elbow crease (EC) that is perpendicular to the wrist crease has been defined as the positive y-axis, as shown in Fig 1. The positive electrode was placed over the elbow olecranon, while the negative electrode was placed at each grid point in order.

#### Stimulation parameters

For all participants, each grid point in a coordinate system was electrically stimulated using current pulse trains with a train duration of 1 s. The pulse frequency and the pulse width of a single pulse were 50 Hz and 400 μs, respectively. A stimulation applied at 50 Hz can achieve a tetanic functional motor response [26–28]. Pulse durations closer to 400 μs produce greater cross-sectional activation [29]. Previous studies have shown that the pulse duration of 400~600 μs and the pulse frequency of 30~50 Hz appear to be the most effective parameters to selectively target motor fibers, thereby minimizing discomfort, muscle fatigue and muscle damage [28–29]. The monophasic stimulus pulses with fixed parameters were not applied repeatedly in the same location in a short time and thus did not cause the large amount of charge aggregation that would lead to human damage [30].

### Experimental process

During the experimental sessions, each participant sat comfortably in a chair with her/his elbow kept in a state of relaxation and free of any constraints, while the forearm and wrist were supported by a height- and direction-adjustable bracket and a rectangular sponge block, respectively. In addition, the angle between the forearm and the upper arm was maintained at approximately 140°. Before initiating a formal program, each participant was given practice trials to ensure that they could perform an individual finger extension/flexion movement. The participants were then given a detailed description of the entire procedure and of the duration of the experiment. The experimental protocol consists of two main parts. The first part is the process of determining the stimulation point: all stimulation points were identified when finger extension/flexion in 3 different forearm postures could be activated. The second part comprises the determination of the activation threshold and selective current ranges of the selected stimulation point for eliciting individual fingers in different forearm postures, along with the analysis of finger angles produced by the selective activation of fingers with different activation thresholds within the selective current range. The total experimental process, excluding the rest time, took approximately 5 hours.

**Fig 2:**
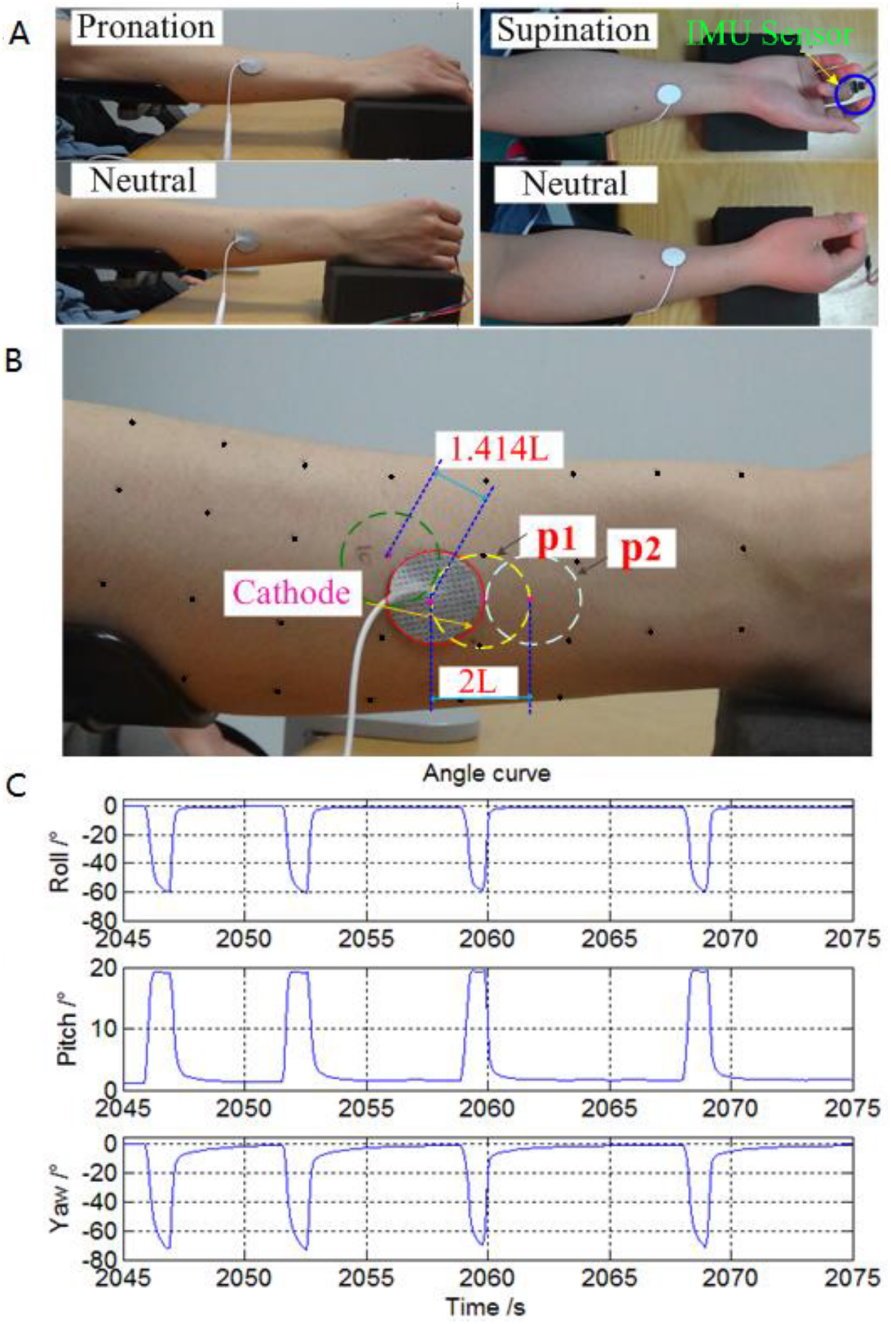
Stimulation points were affected by two factors and its corresponding finger angles. (A) The effect of stimulation point displacement relative to the skin on the finger activation threshold and the selective range of each individual finger were investigated. (B) The negative electrode was placed at distances of 0L, 1L, 1.414L, and 2L away from the center of the original electrode to study whether different electrode positions caused the changes in the activation threshold and selective activation range of individual fingers. (C) The IMU sensor module was used to record the 3D finger movement angle data.

When the forearm was pronated 180°, the stimulation point identification for finger extension in the dorsal side of forearm was conducted. Then, the forearm was pronated 90° while keeping the wrist at 0° (radial and ulnar) deviation, and stimulation points for finger extension in the dorsal sides of the forearm and for finger flexion in the ventral side of the forearm were determined. Finally, the stimulation points for finger flexion were identified in the ventral side of the forearm while the forearm was pronated 0° (Fig 2A).

#### Scanning the grid points and marking the active stimulation points

The scanning procedure began at the coordinate origin in the forearm coordinate system and was performed once in each forearm posture for each participant. The negative electrode was initiated with an 8-mA current intensity at each grid point. This current intensity was chosen to elicit more digit movement after a pilot test across each participant, avoiding more discomfort resulting from the use of a stronger current. As shown in Fig 2B, the negative electrode was moved over the grid points. When the electrode was moved to the next adjacent grid point from one point (e.g., from p1 to p2), there was partial overlap in the coverage regions of these two electrodes. Each grid point was stimulated twice to confirm which fingers were in motion and which type of motion was occurring. Each participant was asked to accurately report any uncomfortable sensation in the testing process. The grid points without pricking, muscle pain, or discomfort were finally marked as stimulation points.

#### Determination of activation thresholds and acquisition of finger movement angles

After the completion of stimulation point identification, we selected some of the stimulation points and tested in their respective two different forearm postures (e.g., stimulation points for finger extension were examined in the forearm pronation and neutral postures) according to the number and the type of the activated fingers and the strength of finger action response to the electrical stimulation at the stimulation point, because it would be a time-consuming task, if each stimulation point of finger extension/flexion identified in the coordination system was used to be investigated. For each selected stimulation point, the activation thresholds of all activated fingers in different forearm postures were determined. The threshold was determined by changing the current intensity applied to the stimulation point, which was first decreased in steps of 1 mA and then adjusted in small steps of 0.1 mA if the finger angle changed slightly with the varying current intensity. When visible finger twitching or minimal movement was observed visually and verified by the PC software, the current intensity applied to the stimulation point was defined as the activation threshold of the stimulated finger. The current range between the activation thresholds of the 1^st^ and 2^nd^ activated fingers at each stimulation point was considered the selective current range of the 1^st^ activated finger (see details in point 1 of section 2.4).

After determining the activation threshold and selective current threshold for the 1^st^ activated finger at each stimulation point in different forearm postures, the stimulus points were divided into 3 groups (“Low”, “Medium” and “High”) according to thresholds of the 1^st^ activated fingers. Integer mA intensity electrical stimulation within the selective current range of the 1^st^ activated finger was applied to each stimulation point. During the testing process, in addition to the complete relaxation of each participant, the stimulation was applied repeatedly to exclude the voluntary and reflexive components of the movements. Each selected stimulation point was repeatedly stimulated 8 times, with a relaxation period of ≥ 5 s between contractions (Fig 2C).

The angle data that the finger produced within the selective current range were recorded by the IMU sensor module and transmitted through the serial port to the PC software.

A custom-written MATLAB script (The MathWorks, Natick, MA) was used to address the angle data stored by the PC software. Three or four active angles were selected within 8 recorded finger angles, the mean value of which was calculated for further analysis.

### Data analysis

#### Selective current ranges of individual fingers

**Fig 3:**
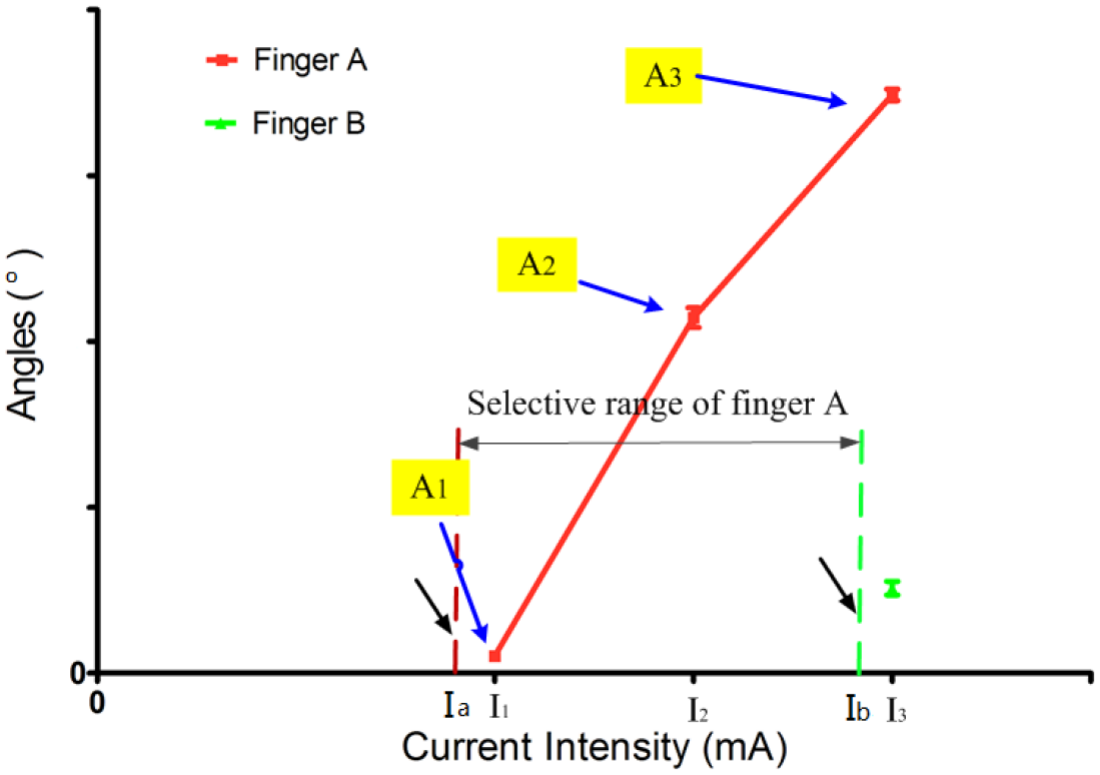
The angle curve of finger A in its selective current range at a stimulus point. Note: T_a_, T_b_: Activation thresholds; *I*_1_ = *ceil* (Ta); *I*_2_ = *I*_1_+ 1; *I*_3_ = *I*_1_ + 2.

The selective current range of each individual finger was defined as the range of current intensity under which only one finger responded to the FES. These ranges demonstrated how selectively a single finger could be stimulated by adjusting the current intensity. Fig 3 shows an example of a stimulation point for which finger A responded to the FES first (at ***I***_a_ mA), followed by finger B (at ***I***_b_ mA), resulting in a selective stimulation current range for finger A of ***I***_a_~***I***_b_ mA. The value of ***I***_1_ was a current intensity equal to the result of function *ceil* (*Ia*) that rounded the threshold Ta to the nearest integer toward infinity. The values of ***I***_2_ and ***I***_3_ were equal to ***I***_1_+1 mA and ***I***_2_+2 mA, respectively. The Tb in Fig 3 was not available for the thumb because of its wide selective current range. The definitions of A_1_, A_2_, and A_2_ are given in point 2 of this section.

**Fig 4:**
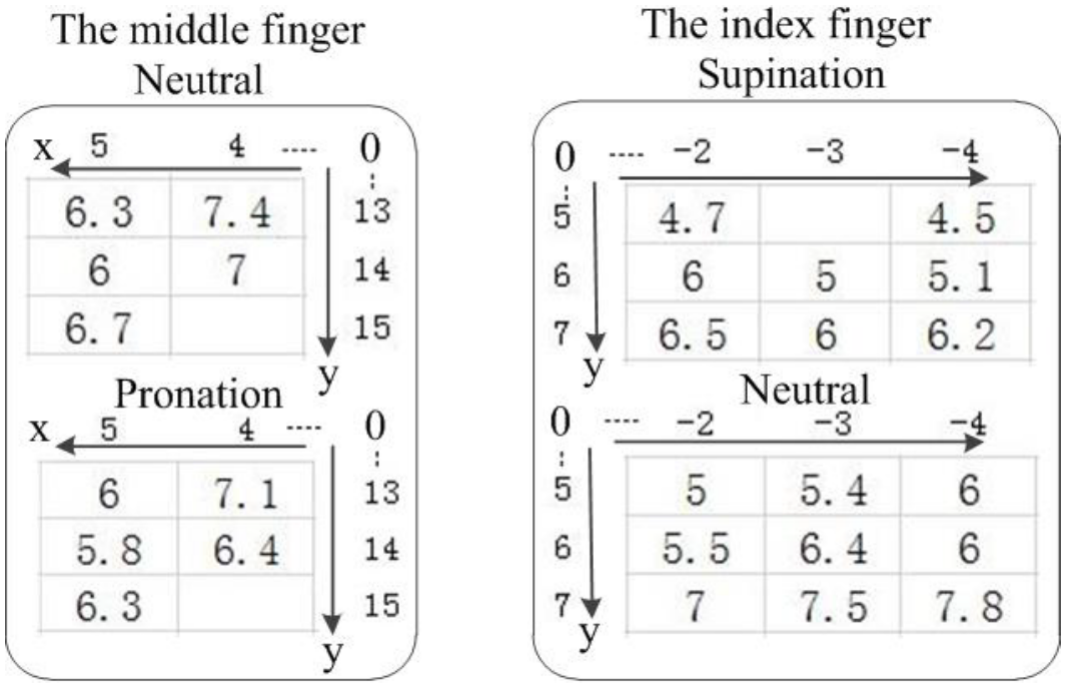
Activation thresholds of several adjacent stimulus points under different forearm positions (Participant D).

The activation threshold of each activated finger could be distinct at two adjacent grid points with a distance of *L*. In addition, as shown in Fig 4, due to the changes in the stimulation point position caused by the relative displacement of the skin and muscles, forearm rotation could change the activation threshold for a specific finger that was always activated at the same grid point. The effects of small changes in the electrode placement and relative displacement of the stimulation point (resulting from forearm rotation) on the threshold amplitude and selective range of each individual finger are described in Section 3.2.

#### The trend of finger angle variation in different sets of activation thresholds

On the basis of the threshold measurements described above, we defined “Low” activation thresholds as those with values of < 4 mA, “Medium” activation thresholds as those with values in the range of 4–6 mA, and “High” activation thresholds as those with values ≥ 6 mA. The angles of each selectively stimulated finger were measured for stimulation points in each threshold group at current intensities of ***I***_1_, ***I***_2_, and ***I***_3_ mA. The trend of finger angle variation of each stimulation point was deduced by observing the slope of each finger angle. As shown in Fig 3, **A**_1_, **A**_2_ and **A**_3_ were the angles produced by finger A at 3 different current levels, ***I***_1_ mA, ***I***_2_ mA and ***I***_3_ mA, respectively.

### Statistical analysis

A 4 × 2 (electrode position × forearm position) two-way ANOVA with Scheffe’s test (*p* < 0.05) was performed to examine the effect of the electrode position change and relative shift of stimulation points on the activation threshold and the selective range of each individual finger. A Kruskal-Wallis one-way ANOVA was used to analyze the effect of the finger activation threshold on the trend of angle variation of each finger at increasing current intensities.

## Results

### Determination of stimulation point distribution for finger extension and flexion

**Fig 5:**
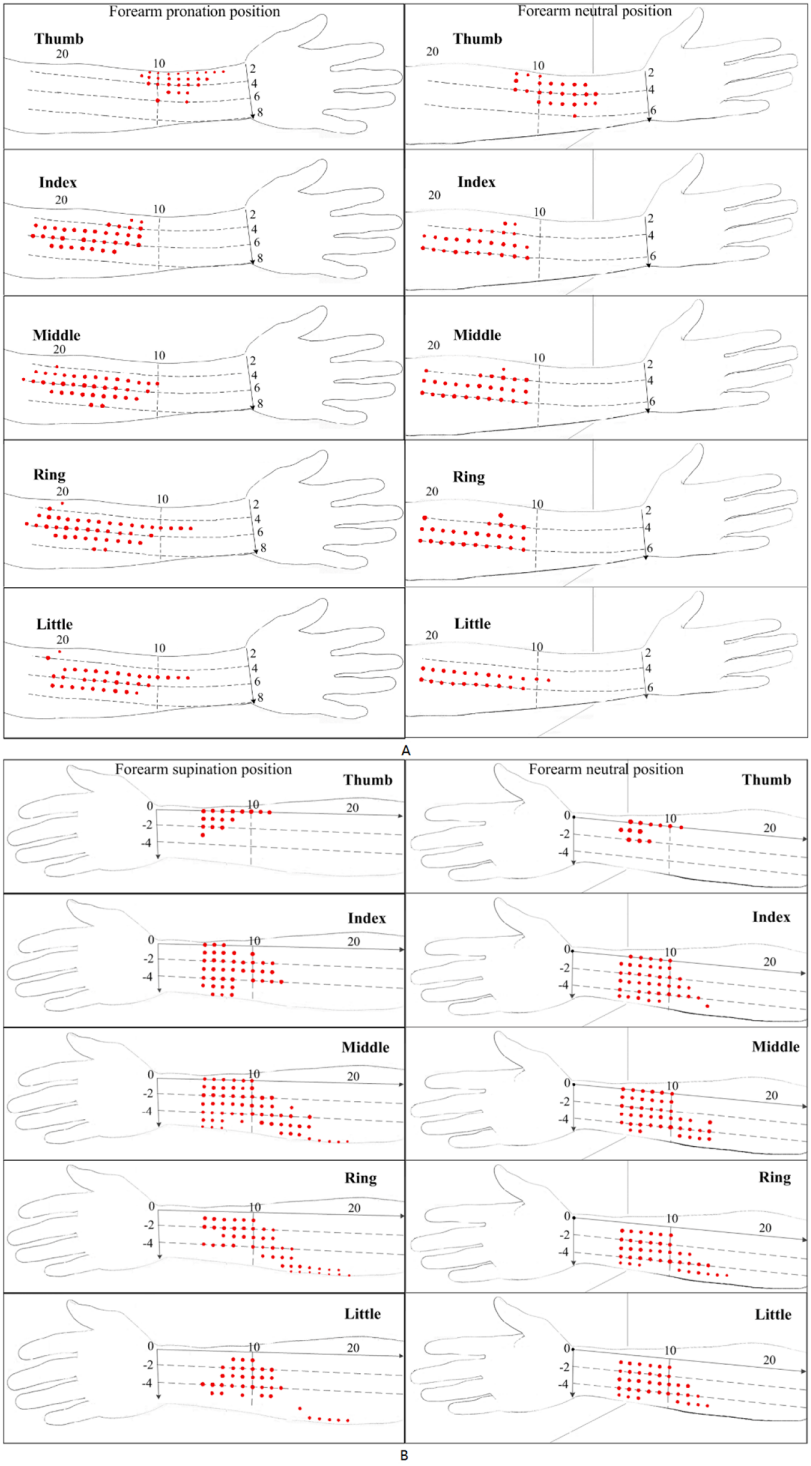
The positions of stimulation points for extension and flexion of each finger. (A) Finger extension in the forearm pronation and neutral positions; (B) finger flexion in the forearm supination and neutral positions.

The length of forearm can be divided into 12 equally spaced segments from the elbow crease to the wrist crease. This method contributes to the location of stimulation point for the finger extension/flexion. Fig 5 shows the positions of stimulation points for extension/flexion of each finger (for participant D), which were identified using 8-mA electrical stimulation at each of the grid points in the forearm coordinate system. When the forearm was rotated from pronation or supination to the neutral position, the grid points that corresponded to the muscle stimulation points in the original forearm position for finger extension or flexion moved toward the radial side of the forearm. By contrast, the muscle stimulation points themselves moved toward the ulnar side of the forearm. In the different forearm postures, both the number of stimulus points for each finger and the size of the stimulation point distribution were changed. For digits 2 to 5, most of the stimulation points were in the same position on the forearm.

**Fig 6:**
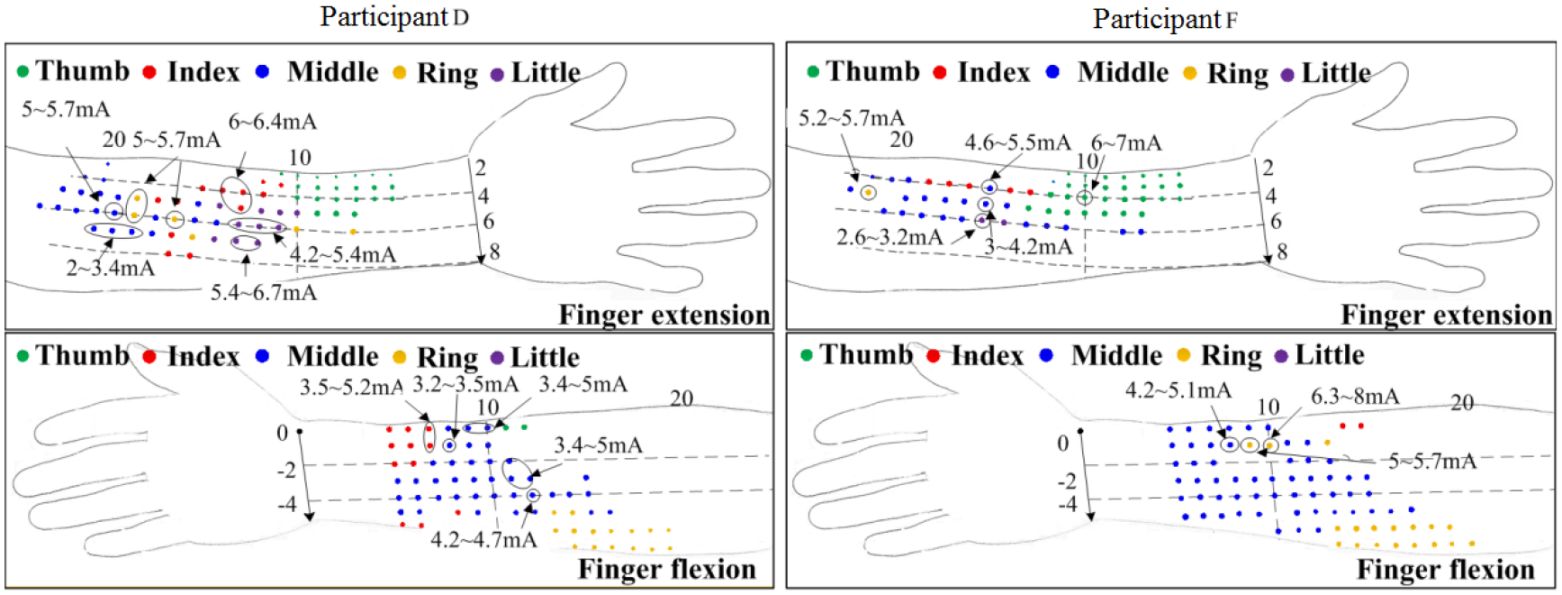
Distributions of stimulation points for selective stimulation of each finger extension (top) and flexion (bottom). Some stimulation points are surrounded by circles of different sizes. The selective ranges of stimulation points for selectively stimulating individual finger extension/flexion were measured by adjusting the intensity of the applied current.

Fig 6 shows the maps of distributions of stimulation points to selectively stimulate extension/flexion of individual fingers in two forearm positions (pronation and supination) for participants D and F. Points in different colors indicate the positions of the stimulation points that selectively stimulated each finger. There was inter-participant variation in the location of the stimulus points for selective activation of the same finger, such as the stimulation point positions for selective stimulation of ring finger extension and index finger flexion. The same grid point among the participants could selectively stimulate different fingers, such as stimulation of finger extension at position (5, 10). We measured the selective current range for several stimulation points that selectively activated a single finger movement and used arrows and differently sized black circles to mark these stimulation points in Fig 6. Different stimulation points that selectively activated the same finger required different threshold current amplitudes and selective ranges. Only a small portion of stimulation points could activate the same finger within the same current intensity range. Both participants were able to selectively activate extension of all fingers. For finger flexion, participant D could selectively activate 4 fingers, whereas participant F could selectively activate only 3 fingers.

### Activation threshold and selective range of individual fingers

**Fig 7:**
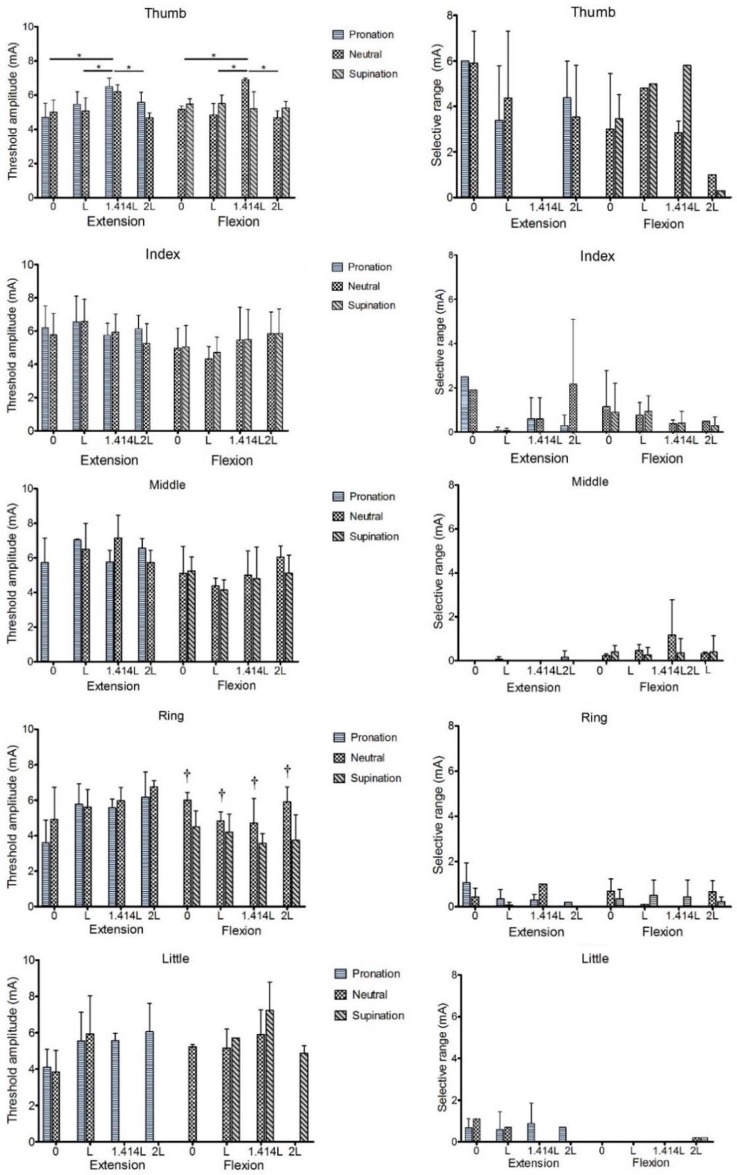
Activation thresholds (left) and selective ranges (right) of individual fingers. (*) represents a difference in the activation thresholds at different electrode positions. (†) indicates a significant difference in the selective activation thresholds of fingers at the same grid point in different forearm positions. The results are shown as the means ± SD (*n* = 8). *^,^ † *p* < 0.05 as determined by two-way analysis of variance.

Fig 7 shows the activation threshold and selective range of individual fingers’ extension/flexion at 4 adjacent electrode positions in different forearm postures. For the thumb, the current intensity required to selectively activate the extension/flexion at grid point 1.414*L* (Fig 2B) in distance from the original grid point was greater than those at any other adjacent grid points (*p* < 0.05). In the neutral and supination forearm positions, the relative displacement of the stimulation point significantly affected the selective activation threshold of the ring finger (*p* < 0.05). In the forearm pronation posture, some stimulation points could selectively stimulate extension of the little finger, but not at some locations in the forearm neutral posture.

Small changes in the electrode position in a specific forearm posture and forearm rotation had no significantly effect on the selective range of each individual finger (*p* > 0.05). However, the selective range of each individual finger showed some dependency on electrode position and forearm posture. For instance, the selective range for the thumb was absent at a certain electrode position or reduced in one forearm posture relative to another. For all participants, the thumb had the largest selective current range, followed by the index and ring fingers. The current ranges for selective activation of middle finger flexion and little finger extension were limited and smaller.

### The influence of forearm position on finger joint angles

**Fig 8:**
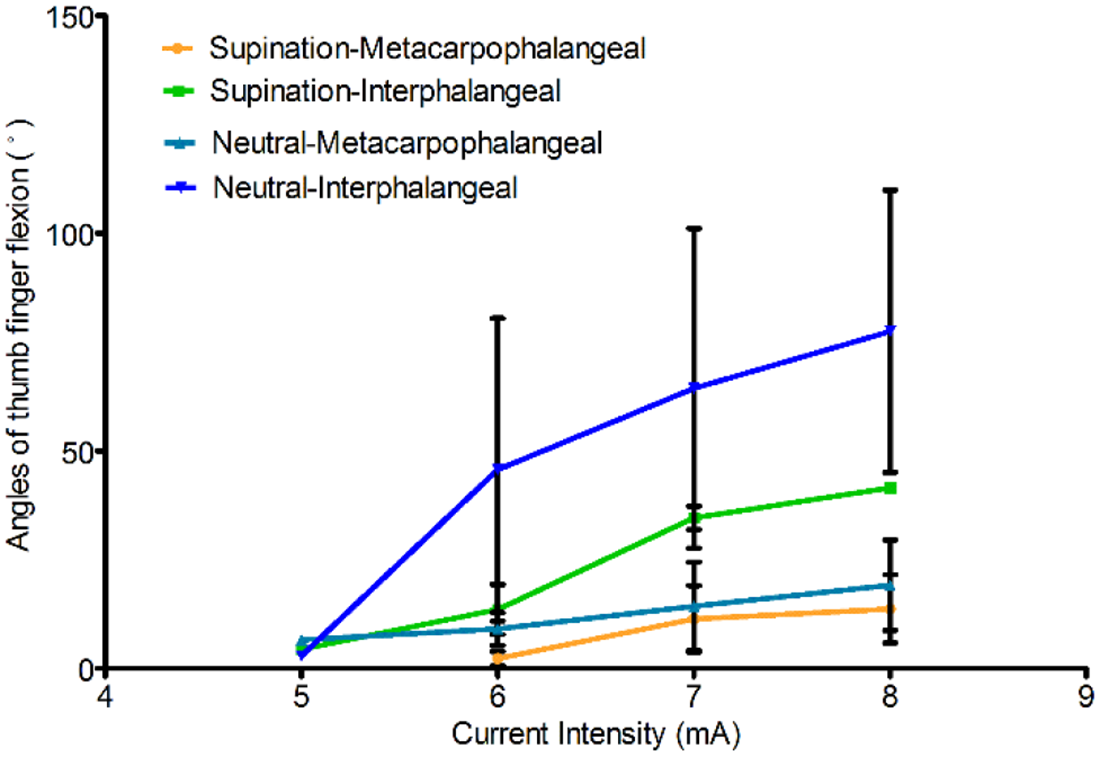
Trends of angle variations of the thumb MCP and IP joints. The angles were tested in supine and neutral forearm postures.

Fig 8 shows the angle variations of the thumb in the forearm supination or neutral position. The IMU sensor module was mounted between the MCP and IP joints to record the angles of the thumb MCP joint and between the IP joint and the fingertip to monitor the angles of the IP joint (not the net angle defined as the MCP joint angle minus the IP joint angle). With an increase in the current intensity, the angle of the thumb MCP joint changed slightly, whereas the angle of the IP joint changed significantly. Therefore, in the next section, the effects of the activation thresholds on the angle of thumb movement were analyzed using the angle of the thumb IP joint.

### Angle variation trends of each activated finger

**Fig 9:**
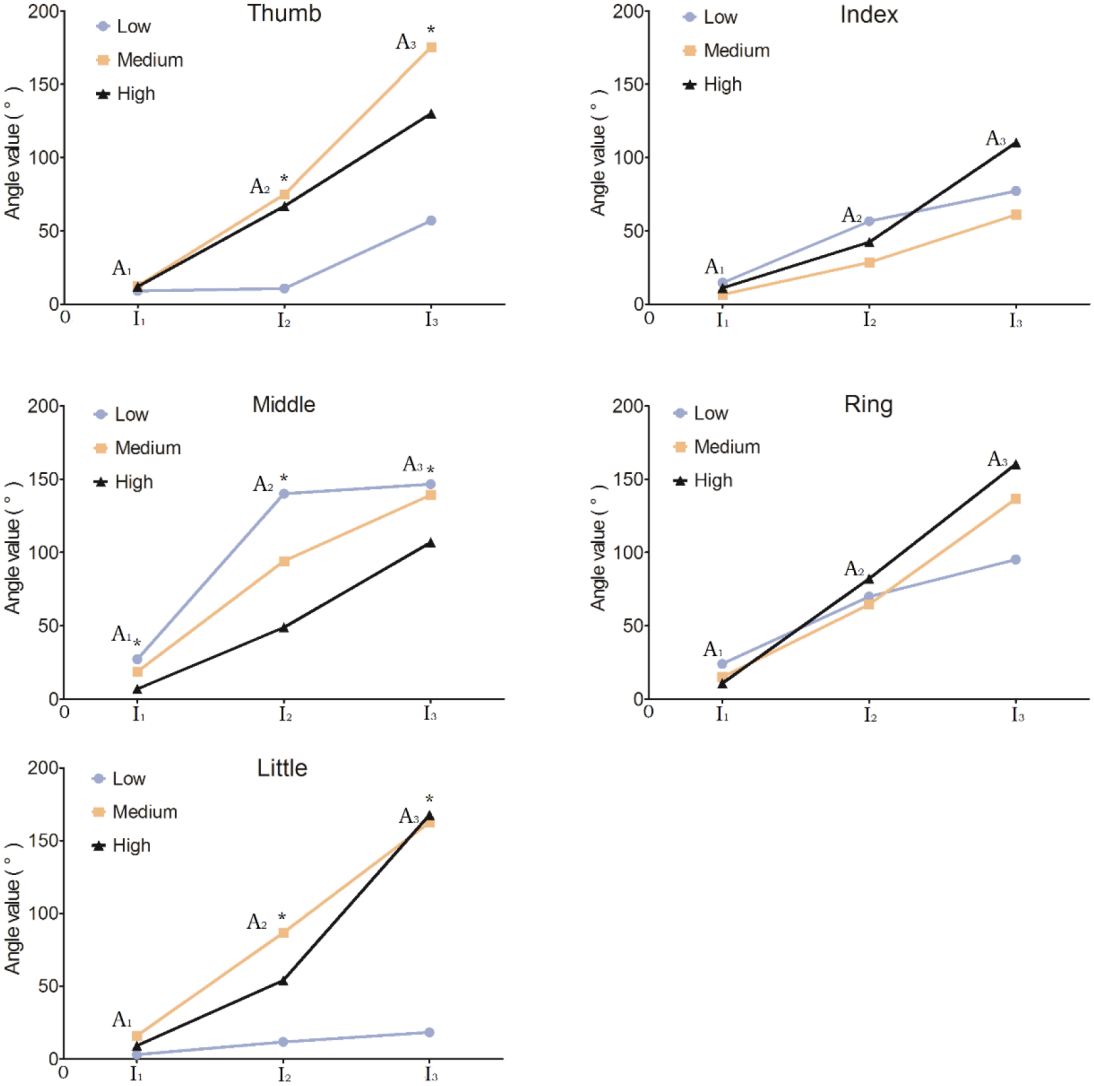
The angle variation trends of the stimulation points in the "Low", "Medium" and "High" groups. The results are expressed as the means (*n* = 8). *p < 0.05, as determined by one-way ANOVA.

The results in Section 3.2 showed that the selective range of the thumb was greater than 2 mA, whereas the selective ranges of the other 4 fingers were less than 2 mA. Thus, for the 2^nd^ to 5^th^ fingers, the currents of ***I***_2_ and ***I***_3_ (defined in Fig 3) were no longer selective. To explore whether the activation threshold of the stimulation point could influence the angle variation of the stimulated finger, we assumed that the current intensity at which the stimulation point produced the angles **A**_2_ and **A**_3_ was still selective. Fig 9 shows the angles of the stimulated finger induced by the stimulation points in three groups. For the thumb and little finger, the initial angles of **A**_1_ in the “Low” group were the smallest (*p* < 0.05). When increasing the current intensity, the finger angles caused at the stimulation points in the “Middle” and “High” groups were significantly larger than those in the “Low” group. The slopes of the angle variation were greater than those in the “Low” group. For the index and ring fingers, the stimulation points in the “Low” group produced the largest initial angles of **A**_1_. As the current intensity increased, the slopes of finger angle variation for the “Low” group became gradually smaller than those for other groups. The stimulation points for the middle finger in the “Low” group produced the largest finger angles at the current intensities ***I***_1_ and ***I***_2_ (*p* < 0.05). The middle finger angles of **A**_3_ at the current intensity of ***I***_3_ in the “Low” group were significantly larger than those in the “High” group (*p* < 0.05). As the current intensity was increased, the slope of angle variation in the middle finger of the “Low” group also gradually became smaller than those of the other two groups.

## Discussion

In this study, we obtained the position and range of the stimulation point distribution for finger extension and flexion in different forearm postures after 8-mA FES was applied to traverse the grid points in the forearm coordinate system. Some of the stimulation points that could activate individual fingers were selected, and they were then tested to obtain the selectively activated fingers and the corresponding selective current ranges. The activation thresholds and selective current ranges of these selectively activated fingers at the tested stimulation points were all determined by reducing the intensity of the applied current. The results showed that the current intensity required to activate a single finger action may be distinct for different stimulation points. Because muscular responses are strongly dependent on electrode position and because of the relative displacement between the skin and muscle resulting from forearm rotation or strong muscle contraction, we investigated the effects of changing the electrode position in a specific forearm posture and the relative shift of the stimulation point on the activation threshold and selective range of an individual finger. As shown in Fig 7, those experiments produced the following results:

1. The thumb activation threshold at an electrode position of $\sqrt{2\text{L}}$ was significantly different from those at other electrode positions;
2. The current intensity required for selective activation of ring finger flexion in the forearm neutral position was much larger than that in the forearm supination position;
3. The required current amplitude for activating a single-finger extension was slightly greater than that required for finger flexion;
4. The selective activation ranges of each finger were different and limited, as the selective ranges for thumb extension and flexion were > 2 mA, and those for the 2^nd^ to 5^th^ fingers were < 2 mA;
5. The index finger had the second-largest activation range after the thumb. The selective stimulation of middle finger extension and of little finger flexion was least achievable via the application of surface FES to the forearm muscles.

It was possible to selectively activate the extension and flexion of most fingers by stimulating the forearm muscles. These results suggested that the use of high-resolution FES to stimulate the forearm muscles was likely to achieve selective activation of individual fingers. The current threshold required to selectively activate most of the fingers varied slightly despite changes in the electrode position and the forearm posture.

We conducted a further analysis (shown in Fig 9) based on the results described above. The stimulation points that could selectively stimulate a single finger had different activation thresholds and were found to have the following characteristics:

1. In the current range for selective activation of an individual finger, the slopes of the finger angle variation in the “Low” group for digits 2~4 were inversely proportional to the current intensity. Meanwhile, the slopes of the finger angle variation in the other groups for digits 2~4 and in all 3 groups for the thumb and little finger were proportional to the current intensity.
2. For the selective activation of the thumb and little finger, the stimulation points with higher activation thresholds resulted in higher finger angles when compared to the stimulation points in the “Low” group (*p* < 0.05). Therefore, a stimulation point with a high activation threshold may be more appropriate for the selective activation of the thumb or little finger to achieve the target action.
3. For the index and ring fingers, as shown in Fig 9, there were no significant differences in the finger angles generated by the stimulation points in the 3 groups at each current level within the selective activation range.
4. The stimulation points for the middle finger in the “Low” group produced larger finger angles than those in other 2 groups over the selective current range (*p* < 0.05).

In a previous study, the location at which a robust muscle contraction could be achieved when the minimum current was injected was called the motor point [30]. As shown in Fig 9, at the small injected current (***I***_1_), the stimulation points in the “Low” group for all 5 fingers produced noticeable movements, especially of the index, middle, and ring fingers. However, the stimulation points in the “Low” groups for 3 or 4 fingers produced smaller finger angles than those of the other 2 groups at the current intensities ***I***_2_ and ***I***_3_. Therefore, we believed that if the electrode placement used for the selective activation of individual fingers was confined to the location with the lowest activation threshold [19], it might reduce the possibility of achieving the target action within the selective current range.

### Physiological aspects

The selective stimulation of individual fingers may result from stimulating a single muscle component through a single nerve branch [10]. The FPL muscle dominates thumb flexion, whereas the extensor pollicis longus (EPL) and extensor pollicis brevis (EPB) muscles induce thumb extension. Thumb extension and flexion are the most independent movements when the thumb muscles are selectively stimulated. The FDS and EDC are superficial multi-tendoned muscles with sub-compartments involved in the selective activation of digits 2~5 [21, 31]. The FDS connects the PIP joint of the finger via tendons, the deeper stratum of which divides into sub-compartments for the index and small fingers and the superficial stratum of which controls the ring and middle fingers. The index finger can also be extended by contraction of the EI muscle. The greater independence of the index finger relative to digits 3~5 can be attributed to the selective stimulation of the EI muscle, and the selective range for index finger flexion may result from the stimulation of the medial nerve innervating the lateral parts of the index finger [22–23]. The coupling of nerves and additional connective tissues between the muscles and the tendons is likely to result in a relatively small selective range of flexion for the middle, ring and little fingers [32]. The middle finger had the smallest amount of independent movement [33]. Our result showed that the stimulation points for selective stimulation of the middle finger were identified in participants D and F (Fig 6). The middle finger extension had the smallest selectivity range (Fig 7), which is likely the result of the inter-participant variation in the stimulation points for individual finger selective stimulation. Lang et al. observed that the neuromuscular coupling was greatest in the control of the ring and little fingers [32, 34]. Extension of the little finger can be obtained by activation of the EDM muscle [18]. The flexion of the little finger has the smallest selectivity range, which was similar to that of middle finger extension. Our study was consistent with the previous report that functional connection from the FDS muscle to the little finger is missing in almost 60% of examined participants (Fig 6) [32].

No significant differences in the threshold amplitude and the selective ranges of most fingers were observed, though the finger activation thresholds changed between adjacent electrode positions, and stimulation points also shifted relative to the skin surface during forearm rotation (Fig 7). For the thumb, the electrode position or the intensity of the current should be adjusted to achieve the activation threshold, given that a relatively large displacement between the muscle and the skin was observed during forearm rotation. For other fingers, when the forearm posture was changed, only a slight increase in the current intensity was needed to activate the target finger.

Low levels of electrical stimulation can easily activate motor units (MUs) with lower stimulation thresholds that are close to the stimulation electrode. These may be primarily the fatigable type II-B MUs. Then, the fatigue-resistant type I and II-A MUs with higher activation thresholds and deep II-B MUs begin to be activated with increasing in FES intensity [20]. Fig 9 shows that the slopes of the finger angle variation in the “Low” group for digits 2~4 were inversely proportional to the current intensity. This may be because a subset of activated MUs of type II-B underwent fatigue. We speculated that the stimulation points with higher activation thresholds might activate more MUs of types I and II-A on the basis of the change in the slope of the finger angle.

Due to the neuromuscular coupling of the nerves and muscles and the connections of the tendons, the independent range of movement of a single finger is limited. Thus, the physiological characteristics of each finger should be considered if the user wants to achieve the selective stimulation of an individual finger through surface FES. For instance, it is suggested that the locations with a lower activation threshold are more appropriate for selective stimulation of the middle finger because it is the least independent [33].

### Related works

Keller et al. succeeded in achieving selective flexion of most of the fingers but were unable to selectively stimulate the little finger [24]. Our results on this point are nearly identical to those of Keller’s study (Fig 7). Nathan [34] found that the thumb can be selectively stimulated by the FPL and thenar muscles, whereas Westerveld et al. observed that selective stimulation of thumb flexion and middle finger extension was possible. They also found that selective stimulation of the index and ring finger was possible in most cases, and 3 or 4 fingers could be selectively stimulated for most participants [10]. Our results showed that selective stimulation of the forearm muscles can be used to induce extension and flexion of most of the fingers, with the exceptions of middle finger extension and little finger flexion. The fact that the single-tendoned muscles that control thumb extension and flexion, such as the FPL, EPL, and EPB muscles, are located in the forearm might explain the finding that the thumb possesses the largest selective range.

Lawrence et al. investigated the movement trajectory of the motor points relative to the skin surface during forearm rotation [21]. They observed that the change in position of the optimal motor points for selective finger flexion was up to 4 cm, whereas the locations of optimal motor points varied relatively little for finger extension, thumb flexion and thumb adduction. We investigated the changes of the activation threshold and selective current range of the selectively activated finger for the same grid point that was stimulated during forearm rotation. As shown in Fig 7, the shift of the stimulation point position relative to the skin surface did not significantly affect the activation thresholds and selective ranges of the selectively activated finger. That is to say, once the activation threshold and selective current range of an individual finger are determined at a certain location, it is not necessary to greatly adjust the intensity of the applied stimulating current if there is a slight change in the forearm posture of the participant. This finding is favorable for the use of surface FES to achieve fine finger control. We found that the current amplitude required for activating the extension of a single finger was slightly greater than that required for flexion of a single finger. The positions and distributions of the stimulation points for selective stimulation of individual fingers were similar among participants, which would be helpful for rapid and accurate placement of the electrodes in laboratory settings. The results in angle variation trends of stimulation points with different activation thresholds provided us with theoretical guidance to successfully and selectively stimulate a single finger.

De Marchis et al. utilized a surface array with fixed electrode positions, containing multi-electrodes with stimulation (negative) and return (positive) contacts, to identify the optimal stimulation point for hand opening [35]. They applied single pulses with a pulse width of 500 μs at three different amplitudes (9 mA, 10 mA and 11 mA) to the stimulation patterns (defined as a pairs of negative and positive electrode) without inducing discomfort, while the surface electromyography (sEMG) signal generated by the stimulated muscle during the pulse was recorded. After removing lower-frequency movement artifacts, high-frequency noise, and stimulation artifacts, sEMG signals contained only the muscular response to the applied stimulation, corresponding to the evoked M-wave. The optimal stimulation pattern for a muscle was determined by comparing the peak-to-peak length of the median M-wave. Then, a 5-s FES burst with a frequency of 30 Hz and a pulse width of 500 μs was carried out to examine the robustness of the optimal stimulation pattern during forearm rotation. This may indeed be a novel method used for determining the optimal stimulation pattern. However, a series of complex signal processing algorithms must be conducted to obtain the muscle response (M-wave). It was not stated whether the frequency of the single pulse used was more than 20 Hz, because FES below 20 Hz cannot induce a fusion contraction. Moreover, the authors also proposed that the method used might be particularly useful for those severely affected patients who show no residual hand movement at all, given that the muscle spasticity would limit the determination and detection of any muscle twitches. In our study, we used a 1-s FES pulse sequence with a current level of 8 mA, a pulse width of 400 μs, and a frequency of 50 Hz to scan the grid points in the forearm coordinate system. Our study provided the electrode placement position for selective stimulation of individual fingers in different forearm postures. We further investigated how the activation threshold and selective current range of the selectively activated finger varied due to changes in the electrode position and forearm rotation and whether the stimulation points with different thresholds produced the same finger angle variation within the selective current range. Compared to De Marchis’s study, we identified all the stimulation points that induced individual fingers, rather than the optimal stimulus pattern. In addition, the methodology used in our study did not involve complex processing algorithms, making it easier to conduct.

### Limitations

In this study, experiments were performed only in healthy human participants. We achieved the purpose of selectively activating individual fingers by applying electrical stimulation to the forearm muscles. During the experiments, we found that some stimulus points could elicit both the finger and the wrist extension/flexion at higher current intensities, and some stimulus points induced both the thumb extension and wrist abduction. However, the wrist joint kinematics were not recorded during the testing process because this point was out of the scope of the study.

In the future, factors that affect muscle selectivity should be taken into account as much as possible, and the experimental design should also be extended to stroke patients. Although paralyzed patients show changes in muscle properties, the skin and the underlying muscles that constitute the geometry of the forearm change only slightly [13, 15]. This geometry is an important factor in the spatial selectivity of surface stimulation [10]. We believe that the results of this study will be likely to provide guidance for future clinical trials.

An appropriate method is required to identify and locate the inter-participant variation in the distribution of the stimulation points for the selective stimulation of finger extension and flexion, resulting in a personalized stimulation. Future methods designed for fine finger control should include a high-density array of electrodes and should have the ability to locate the appropriate stimulation points in participants with reference to the present results according to the needs of the functional task to be completed.

There was no design to measure subjective comfort in our study. Stimulation was stopped when participants reported unbearable discomfort. However, we found that most participants were able to tolerate stimulation at 8 mA. In theory, a greater stimulation intensity would result in a different distribution of stimulation points for finger extension/flexion, that is, each stimulation point would activate more fingers than the lower current intensity. However, the selective activation of a single finger at 8 mA of electrical stimulation was impossible in most participants because multiple fingers were activated. Thus, 8 mA of electrical stimulation was enough for most cases.

### Practical applications

The results of this study show the positions at which we can place a negative electrode or an electrode array for the selective stimulation of finger extension/flexion in different forearm postures. The small differences in the activation threshold and selective range between different selectively activated fingers indicate that a high-resolution stimulator for the fine control of stimulus current and pulse duration, like the Master-9^™^ stimulator, should be designed. High-resolution stimulation could enable stroke patients who have been able to achieve gross functional hand opening to perform tasks requiring fine finger control. Fig 6 shows the stimulation points that selectively activated single-finger extension/flexion for two participants. Although the number and location of the stimulation points for selectively activating individual fingers differed slightly, the positions of the stimulation points for finger extension/flexion were generally consistent relative to the length and width of the forearm.

FES induces muscle contraction in a non-physiological way, which may not follow the size principle and may recruit motor units in a nonselective manner. Thus, in practical applications, early fatigue may still occur due to the stimulation to identical muscle fibers. As shown in Fig 6, the stimulation points for selectively activating a single finger were not unique. Changing the applied stimulus strategy [36] or providing asynchronous stimulus [37] of stimulus points may be an effective way to alleviate muscle fatigue.

At present, a wearable multi-pad-based prototype for selective FES has been developed in our group [38]. In future, we intend to develop a specific electrode array based on the shape and distribution of stimulation points detected in this study for selective stimulation of finger extension/flexion. Then, an iterative learning algorithm and sensor feedback may be used to identify the stimulation points that selectively activate individual finger and to control stimulating pulse output [39–40]. The frontend circuit of the stimulator should be further improved on the basis of the multi-pad wearable selective FES so that the current output resolution could be consistent with the research requirements.

## Conclusions

The results of this study show that it is possible to selectively stimulate individual fingers. However, the current range of this selective stimulation varies among the fingers. A small change in the position of the electrode in a specific forearm posture and the relative displacement of the stimulus point affected the activation thresholds and selective range of most fingers, but this effect was not significant. The high density of the electrode array and high-resolution stimulation should be taken into account while applying electrical stimulation to the forearm muscle for selective stimulation of a single finger. The electrode placement used for the selective activation of an individual finger should not be confined to the location with the lowest activation threshold. The results presented here may contribute to the development of equipment for implementing fine control of finger movement in clinical trials.

## Supporting information

S1 Appendix The raw dataset of finger extension and flexion angle for 8 subjects. The Excel files are the raw data collected relating to the grid points’ response to surface FES and the initial finger angle data analysis.
(RAR)
